# The effect of paramedic training on pre-hospital trauma care (EPPTC-study): a study protocol for a prospective semi-qualitative observational trial

**DOI:** 10.1186/1472-6920-14-32

**Published:** 2014-02-15

**Authors:** David Häske, Stefan K Beckers, Marzellus Hofmann, Christoph G Wölfl, Bernhard Gliwitzky, Paul Grützner, Ulrich Stöckle, Matthias Münzberg

**Affiliations:** 1 Faculty of Medicine, Eberhard Karls University Tuebingen, 72076 Tuebingen, Germany; 2 Department of Anaesthesiology, University Hospital RWTH Aachen, 52057 Aachen, Germany; 3 Emergency Medical Service, Fire Department, City of Aachen, 52057 Aachen, Germany; 4 Faculty of Health, University of Witten/Herdecke, 58448 Witten, Germany; 5 Department of Trauma and Orthopedic Surgery, BG Hospital Ludwigshafen, 67071 Ludwigshafen, Germany; 6 PHTLS Germany, DBRD-Akademie GmbH, 76877 Offenbach/Queich, Germany; 7 Department of Traumatology and Reconstructive Surgery, Berufsgenossenschaftliche Unfallklinik Tuebingen, University Hospital, Eberhard Karls University Tuebingen, 72076 Tuebingen, Germany

**Keywords:** Paramedic, Pre-hospital, Trauma, Training, PHTLS, Medical education

## Abstract

**Background:**

Accidents are the leading cause of death in adults prior to middle age. The care of severely injured patients is an interdisciplinary challenge. Limited evidence is available concerning pre-hospital trauma care training programs and the advantage of such programs for trauma patients. The effect on trauma care procedures or on the safety of emergency crews on the scene is limited; however, there is a high level of experience and expert opinion.

**Methods:**

I – Video-recorded case studies are the basis of an assessment tool and checklist being developed to verify the results of programs to train participants in the care of seriously injured patients, also known as “objective structured clinical examination” (OSCE). The timing, completeness and quality of the individual measures are assessed using appropriate scales. The evaluation of team communication and interaction will be analyzed with qualitative methods and quantified and verified by existing instruments (e.g. the Clinical Team Scale). The developed assessment tool is validated by several experts in the fields of trauma care, trauma research and medical education. II a) In a German emergency medical service, the subjective assessment of paramedics of their pre-hospital care of trauma patients is evaluated at three time points, namely before, immediately after and one year after training. b) The effect of a standardized course concept on the quality of documentation in actual field operations is determined based on three items relevant to patient safety before and after the course. c) The assessment tool will be used to assess the effect of a standardized course concept on procedures and team communication in pre-hospital trauma care using scenario-based case studies.

**Discussion:**

This study explores the effect of training on paramedics. After successful study completion, further multicenter studies are conceivable, which would evaluate emergency-physician staffed teams. The influence on the patients and prehospital measures should be assessed based on a retrospective analysis of the emergency room data.

**Trials registration:**

German Clinical Trials Register, ID DRKS00004713.

## Background

Accidents are the leading cause of death in adults prior to middle age [1]. A patient collective is concerned which could particularly benefit from rapid recovery after an accident. In addition to the personal suffering and loss of so-called human capital (in work and leisure activities), the economic costs are relevant and have an effect on the gross national product [2]. The economic costs of traffic accidents in Germany amount to 31 billion euros per year [3].

In recent years, there have been a number of indications of the importance of study findings concerning trauma care, including the following: 1) the introduction of regional trauma-networks [4,5]; 2) a White Paper from the German Trauma Society [6,7]; 3) increased training of emergency department teams [8] and training programs in emergency medical services [9]; 4) the establishment of the TraumaRegister DGU® of the German Trauma Society [10]; and 5) the German S3 – Guideline on Treatment of Patients with Severe and Multiple Injuries (2011) [1].

In the 1970s, “Advanced Trauma Life Support” (ATLS) introduced standardized treatment of trauma patients in emergency departments. Probable total of approximately 2 million physicians are trained in the program worldwide [11]. The global ATLS program is a condition for certification for trauma centers in Germany [12]. Münzberg et al. showed that since 2003, ATLS has performed with high ratings based on evaluation by the participants [11]. Based on the “lowest common denominator”, ATLS is a concept for training the physicians dealing with severe injured patients in the emergency departments in Germany [13]. Ruchholtz et al. showed in the 1990s that procedures based on the guidelines could improve the care of emergency room patients [14]. Evidence of the effect on patient outcome is lacking [15].

The interface between emergency medical services (EMS) and emergency departments in the treatment of seriously injured patients benefits from uniform standards of care based on identical medical-scientific knowledge and communication with a “common language” to avoid errors and to improve priority-based patient care [16].

The treatment of severely injured patients is particularly challenging because these injuries are rare [17] and require multi-disciplinary team work and complex and comprehensive therapy [18]. Pre-hospital care is in parts associated with considerable technical effort.

The pre-hospital care of severely injured patients was essentially characterized in the past by the two following systems: “stay and play” or “load and go” [19]. In recent decades, pre-hospital trauma care has been supplemented by the findings of military medical treatment [20] and appear to approach each other [21].

Pre-hospital treatment has to establish the initial treatment strategies and be priority-oriented. The available data are inadequate, although field experiences and expert opinions are extensive.

The existing trauma care training programs such as the Pre-Hospital Trauma Life Support (PHTLS) or ATLS programs lead to more subjective safety levels of the participants regarding the care of trauma patients [22,23]. The extent of the effect of this training on the quality of the process and especially on the quality of the primary outcomes of modern EMS systems is not clear [24]. The introduction of a training program such as PHTLS in less developed systems results in a measurable change [25]. Studies that evaluated the outcome of patients after rescue and treatment according to the PHTLS standards have shown no significant advantage over currently established principles of trauma care [26,27]. Standardized training programs such as PHTLS are increasingly integrated into the training or education of EMS staff [28,29]; however, the differences in the current standard of care from the recommendations of PHTLS are unknown. A concrete comparison of the PHTLS content with the current guidelines for the treatment of multiple traumas is pending.

The participants evaluate PHTLS courses very positively, although there are no published evaluations. A subjective assessment by the participants was collected after the PHTLS TEAM (PHTLS at medical school) training, which suggests an improvement in the scenario based on the care of severely injured patients [23]. Particularly in the area of “non-technical skills”, various assessment instruments have been developed that assess communication, team interaction, and decision making [30-34].

There is no suitable measuring instrument to ensure the effect of training objectively like the processes, strategic decisions, skills and medical aspects of treatment.

This study will evaluate the effect of PHTLS courses on the participants because its effect on patient outcome as known is not measurable.

### Hypotheses

Using a novel assessment tool (2.4.1), the following hypotheses will be tested:

▪ The introduction of PHTLS leads to improved quality of the documentation of actual field operations.

▪ The introduction of PHTLS leads to structured patient care by the ABCDE scheme [35], with priority-based interventions in case-based scenario training.

▪ The introduction of PHTLS leads the participants to a subjectively better and safer application of the principles.

### Trial design

This trial is designed as an interventional, single arm – uncontrolled, open study. It is a single-center, prospective, semi-qualitative observational trial.

## Methods

### Study setting

The study is performed in the EMS of the city of Wiesbaden and Rhein-Taunus-Kreis (Germany). The operational area in Wiesbaden has five commissioned EMS agencies (four charities, one private provider). The EMS in Wiesbaden has 375 paramedics and serves 462 098 inhabitants.

With regard to the training, close cooperation occurs with the neighboring Rheingau-Taunus-Kreis area. In the context of various difficulties and problems, the controlling authority enabled 301 paramedics from both EMS services to attend the PHTLS courses to create uniform structures and principles [28].

### Eligibility criteria

Included in the study are all the employed paramedics and emergency medical technicians (EMTs) of the participating institutions (ASB, DRK, MHD, private) of the emergency medical services of Wiesbaden and Rheingau-Taunus-Kreis. Excluded are all the participants not involved with the participating institutions.

### Interventions

Paramedical personnel are trained in the care of trauma patients, and the worldwide standardized and certified training program “PHTLS” (Pre Hospital Trauma Life Support) is used.

PHTLS provider courses focus on the professional groups involved in EMS (EMT, paramedics, emergency physicians) for the pre-hospital care of trauma patients and are currently well established in Germany. Among other participants, the helicopter crews of the DRF German Air Rescue will be trained in PHTLS, as well as the German armed forces, which have established PHTLS courses for mission preparation. Thus, PHTLS courses are merged seamlessly with ATLS courses for clinical care in emergency departments [8].

The central link between the PHTLS and ATLS courses is uniform communication and the priority-based approach [22].

In the two-day courses, the participants obtain a complete procedure for the structured treatment of trauma patients, in addition to trauma-specific skills; the classes conclude with a written and practical exam.

PHTLS courses are characterized by the extensive variety in the teaching methods (lectures, practical case studies), with a close instructor- participant ratio (1:3), many practice activities and continuous interaction.

In addition to various skills, the priority-based structure, ABCDE (Airway, Breathing, Circulation, Disability and Exposure), is taught and practiced in scenario-based training sessions. The “ABCDE” method provides structure for patient treatment and ensures that other therapeutic measures are objectified [15].

The PHTLS manual (2^nd^ German edition), which forms the basis for the German PHTLS courses [35], is sent to the participants before the course and is intended to provide the course content with the following priorities:

▪ Safety for EMS staff and patients

▪ Involvement of the accident kinematics in the assessment

▪ Priority-based treatment-principles, “treat first what kills first”

▪ Rapid and correct c-spine immobilization

▪ Immediate repair of airway (A) problems

▪ Evaluation of the ventilation, rapid oxygen administration and treatment of tension pneumothorax

▪ Stop external bleeding and minimization of internal bleeding, e.g. with the pelvic sling

▪ Positioning of axis fractures, and immobilization of the patient if necessary

▪ Treatment of hypothermia

▪ Insertion of iv-lines and fluid resuscitation

▪ Neurological assessment with GCS (Glasgow Coma Scale) and pupil status

▪ Team communications with clear instructions and early clarification to the receiving hospital

As part of the so-called secondary survey, the PHTLS courses provide a SAMPLE scheme with **s**ymptoms, **a**llergies, **m**edications, **p**atient history (including the medical history), the **l**ast oral intake and information about the **e**vent that led to the emergency situation.

The PHTLS courses require a baseline-scenario at the beginning of the course, in which the participants treat a patient in a standardized scenario-based case, without help or feedback from the instructors. The participants obtain an impression of their work before the course. During the second day, a clear shift towards a structured treatment is typically recognized.

### Outcomes

Three outcome measurement methods are used:

#### Assessment tool

The Assessment-Tool has to be used to evaluate objectively the processes and skills during the scenarios. This method is also applied as “objective structured clinical examination” (OSCE) in medical schools [36-39] and used in emergency medical education, too [40,41]. The assessment tool will be designed for the video-based outcome measurement with defined endpoints.

The assessment tool shall take into account three main aspects:

▪ The educational content of the PHTLS courses including oxygen administration, c-spine immobilization and treatment algorithms.

▪ Established assessment instruments such as the Clinical Team Scale (CTS) for the validation of teamwork, team communication and clinical decision making.

▪ Aspects, which are noticeable in the analysis of the scenarios.

Verifiable items are developed from the PHTLS curriculum and from the established measurement instruments, which can be assessed and quantified using an instrument such as the Likert Scale.

Various video-recorded scenarios are used to perform a qualitative content analysis in a non-reactive observation. The relevant features are clustered and transcribed to obtain quantifiable items.

The items are evaluated independently of the measurement scale and summarized as a total score. The fulfilled items will be chronologically registered to analyze the diagnosis procedure and interventions.

Validation of the assessment tools with regard to objectivity and reliability should be based on two scenarios by experts in the fields of emergency medicine, medical education and traumatology. The number of experts will result in a ratio of the items.

The inter-rater reliability, which is the degree of agreement among the experts in the application of the assessment tool, can be verified using Fleiss’ kappa correlation, for which a significance level is set.

#### Questionnaire

The course participants are interviewed with a questionnaire concerning their level of knowledge, skills and safety in the care of trauma patients. These data are collected multiple times and reflect the participants’ subjective assessment of learning success in knowledge, skills and safety. A steady (metric) scale to ± 3 (positive/negative), including zero is used.

#### EMS-operation protocol

As part of the so-called “secondary survey”, the PHTLS courses provide use “SAMPLE” as a mnemonic tool [36]. The letters “AMP” stand for allergies, medication and patient history and are relevant to patient safety [42]. The analysis will review these features in the standardized EMS protocols from actual field operations performed by paramedics, if after the course (not only in trauma patients) the SAMPLE history is used.

The state of Hessen uses the so-called report digits (“Rückmeldezahlen, RMZ”), which report encoded medical indications (e.g. combustion, hyperventilation) and the patient’s condition by a return code “RMC”(Rückmeldecode) and timestamps. The RMC documents a seven-item array consisting of the consciousness, respiration, circulation, injury, neurology and pain of the patient. The minimum number of points of the RMC is six; the maximum number of points is 42. Deviations from the physiological condition of the patient arise from an RMC > 6 points. There is no validation of the correct assessment or use by the paramedics. The extracted identification numbers of the operations are available from the period before the first course started. The identification numbers of the operation-protocols will be randomly selected from the quarter prior to the start of the course. The protocols are selected for the involved EMS agencies proportional to their level of participation in the total operations. The RMC must be greater than 6 points, and selected protocols will be analyzed.

The second analysis is scheduled in the quarter after all of the participants have completed the courses. The frequency of the fulfilled mnemonic is thus compared before and after the implementation of the PHTLS courses.

#### Data from the emergency department

In addition to actually study design, we will evaluate in a retrospective analysis the emergency department data. The investigation is and will be integrated regarding to detailed question and basic circumstances (e.g. ethics application, data privacy). Outcome measurement methods are developed.

#### Bias

In the before-after comparison, the participants know the approach to the patient through other training as well as that taught by the PHTLS. Even reading the PHTLS manual to prepare for the course might influence the participants, as would the fact that with each additional course, knowledge is disseminated by colleagues.

The cohorts of participants are paramedics. Typically, a German EMS crew does not treat seriously injured patients alone because paramedics are supported by emergency physicians in the field. Specific types of invasive skills and analgesia must be excluded from the cases because they are not performed by paramedics in the analyzed area.

##### 

**Mimes** For the baseline scenario, a participant performs the role of the patient; for the case studies, an instructed mime performs a standardized role of the patient. The quality of the representation and the accurate reproduction of specified symptoms might differ under those conditions.

##### Instructors

The vital signs and the non-presentable values are specified by the instructors. Each scenario could be defined differently or be variably stressed. Typically, the scenarios last approximately 10 minutes and are led by the instructors, and the team communication might be limited.

##### 

**Participants** The participants begin the course with different levels of motivation. They should have the identical theoretical knowledge level because they have read the PHTLS manual by the start of the course.

A type of Hawthorne effect may be caused by the fact that the participants know that they are being observed and might be filmed. Regarding the video recording, it is assumed that the more motivated participants offer to be the first team for the scenarios. If the identical participants are also used for the second video recording, the result might not be the result for the study cohort.

##### 

**Technical and organizational aspects** It is possible that the participants did not provide their consent to the recording. Technical problems could affect the video recordings. Organizational difficulties with the equipment (including unpunctual delivery loss of volume) and organizational fault in the recordings (wrong scenario, a bad camera position) might be possible.

#### Timeline

**Figure 1:**
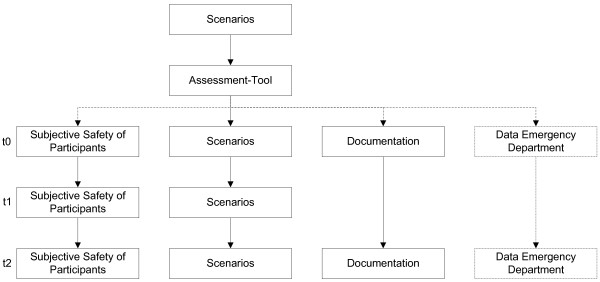
**Project timepoints t0 - t2.** “Subjective Safety of Participants” and “Scenarios” with three time points. Documentation with two time points. “Data Emergency Department” in process of planning.

There are three defined time points for the measurement (Figure 1), as follows:

#### Timepoint t0

Immediately prior to the PHTLS course (pre-course), the participants are interviewed with the questionnaire about their personal judgment regarding their skills, knowledge and safety in the care of trauma patients.

Prior to the start of the training program, the participants in 3-person-team treat a trauma-patient in a standardized case-based scenario, which is recorded on video. Additionally we want to evaluate the emergency department data from 2012.

#### Timepoint t1

At the end of the course, the participants’ survey with the questionnaire and the video recording are repeated to verify the changes (post-course).

#### Timepoint t2

After one year, the participants of the course will be interviewed again with the questionnaire, and the results will be verified by the video recordings in standardized case-based scenarios in practice. Data from the emergency department will be from 2014.

### Number of participants

#### Questionnaire

The number of questionnaires is based on the number of participants. The questionnaires at t0 and t1 will be collected directly, and a 100% return rate is expected. The questionnaire at time t2 will be web-based or distributed via email.

#### Video recording

In the 14 planned courses, 4 video recordings of scenarios completed by three-person teams will be made at t0. A total of 56 teams (168 participants) will be recorded. At t1, video recordings will be made. At this point, three records will be possible, and 12 teams per course will be filmed. In 14 courses, we expected up to 168 teams. The recordings will be made with four camcorders (Panasonic HD Camcorder HC-V100) and recorded on SD Memory Cards.

#### EMS operation protocols

Experts from the Department of Biometry of the University of Tuebingen Hospital (Tuebingen, Germany) recommended the evaluation of 400 protocols to detect changes of approximately 10% at a power of 0,8.

#### Data from the emergency department

pending

### Recruitment

The medical supervisor and the EMS agencies require the participants to attend the training. Study participation is voluntary.

### Data collection methods

#### Questionnaire

The questionnaires at t0 and t1 are distributed and collected during the courses. The survey at t2 (after one year) is conducted using the questionnaires in parallel with the video analysis.

#### Video recording

The videos are recorded at the beginning and end of the training and one year later.

#### EMS operation protocols

The EMS protocols are required in the context of the EMS field operations and archived in the EMS agencies in concordance with the local EMS regulations.

#### Data from the emergency department

pending.

### Data management

The data collection, coding, routing and analysis are coordinated with the data protection officer of the University of Tuebingen and the University of Tuebingen Hospital. The participants will be informed about the study prior to the course, and their questions about it will be answered.

The declarations of consent from the participants for the video analysis are available; the consent declarations assure permission for the recording, analysis and storage of the study data. The number of participants will be registered as a negative figure.

For the questionnaires and time contact, t2 is a declaration of consent for the available participants. This declaration is separated from the questionnaires on site. The questionnaires are pseudonymized with a four-digit code to represent the relationship between the different times, not to establish a connection to the participants.

The analysis of the defined characteristics in the EMS-operation protocols is unrelated to the patient data (including the last name, first name and date of birth). Hessian EMS law § 17 permits the use of evaluation data in the context of quality assurance.

### Statistical methods

The statistical analysis of the end points of the video is based on the assessment tool. The achievable sum scores from the assessment tool are combined with the respective measuring points and, depending on the scale levels, as the mean or median and compared as the independent samples. The statistical tests are dependent on the scale level, and the results are given with the confidence intervals.

The statistical analysis of the questionnaires used the three time points t0, t1 and t2. The individual questionnaires at the respective time points are recorded using Microsoft Excel® 2010 (Redmond, USA). The data are matched in IBM SPSS Statistics 21 software (Illinois, USA) with the pseudonymous ID codes. The data are normally distributed and considered to be metric; the statistical calculation was performed using Student’s t-test for the unpaired two-sided sampling. The significance level is set at α = 0.05.

The evaluations of the EMS protocols in terms of three items, allergies, medication, and the patient’s medical history are conducted by counting the frequency before and after the comparison. The significance level is set at α = 0.1.

### Research ethics approval

The Ethics Committee of the Medical Faculty of the Eberhard Karls University of Tuebingen and the University Hospital approved the study proposal, number 197/2013BO2, on May 24 2013. The study is registered in the German Clinical Trials Register with the ID DRKS00004713.

### Trials status

**Figure 2:**
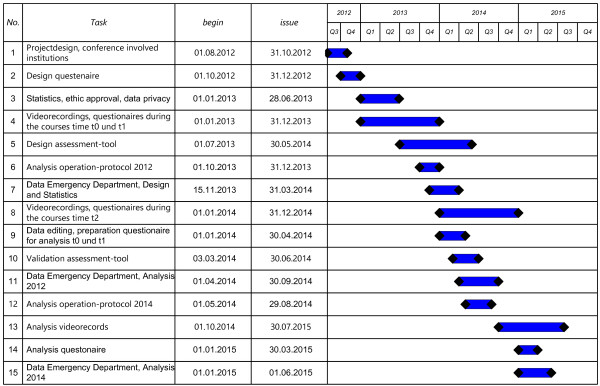
Flowchart of the study, with detailed issues and tasks.

The video recordings as well as the basics of the program and the development of the assessment tools are in process (Figure 2).

## Discussion

The aim to evaluate the effect of the training or the effect of the program on the patient outcome is understandable, complex and difficult. This study attempts to evaluate the effect of the training on the participants’ behavior.

This is a single-center study and based on scenarios of the treatment given to patients by paramedics. There is interest in developing a multi-center assessment tool. Concrete efforts will be made after the completion of this study to apply the tool for evaluation of emergency physician-based teams including air rescue personnel.

We are aware that the video analysis is influenced by a variety of factors. In addition, the result of the scenario-based patient care is not to transfer 1:1 to the real patient care. To increase the comparability and consistency, a member of the research group will supervise the recordings or even leads them through. Time t0 records are parallel, and the scenarios are directed by leading instructors briefed on the key points and identical criteria to apply to all the cases. An identical situation occurs with t1, with the difference being that in t1, the cases are sequential instead of parallel.

Initially it was planned to visualize the individual measures on a timeline. However, the flow in the scenario is disturbed by the information from the instructors, for example vital signs or by questions from the participants, so it does not seem sensible to use the real-time analysis. In addition, a range of skills such as laying intravascular access or prepare infusions are not real performed.

The results of the questionnaire for the subjective assessment of the participants should be available without special bias, especially we expect a very high response rate. The evaluation of the EMS-protocols focuses on three items, which are trained in the course. But since the SAMPLE scheme is widely used, it remains to be seen how the degree of compliance prior to the course and a possible change is measurable.

This study is being extended to encompass a retrospective analysis of the emergency department data because the participants report a significant change in the care of trauma patients in actual field operations. A question remains concerning the degree to which the training of paramedics in PHTLS has an effect on the treatment of trauma patients by emergency physician-supported teams. Key points will be pre-hospital on-scene time, measures and treatment, and any changes in the patient collective. This part is not completed.

## Abbreviations

ASB: Arbeiter-Samariter-Bund, German aid and welfare organization, EMS agency; ATLS: Advanced trauma life support; DRF: Deutsche Rettungsflugwacht, German Air Rescue; DRK: Deutsches Rotes Kreuz, German Red Cross, a non-profit organization, EMS agency; EMS: Emergency Medical Service; EMT: Emergency medical technician; JUH: Johanniter Unfallhilfe, German Aid and Welfare Organization, EMS agency; MHD: Malteser Hilfsdienst, German Aid and Welfare Organization, EMS agency; PHTLS: Pre-Hospital trauma life support.

## Competing interests

DH, MM, CW are PHTLS instructors. BG is the Chairman of PHTLS Germany and Manager of the DBRD-Akademie GmbH. The other authors declare that they have no competing interests. The study is funded by the German Association of Emergency Medical Technicians (Deutscher Berufsverband Rettungsdienst e.V. DBRD). Further fundings are requested. There is no influence in the study design, data collection and analysis, decision to publish, or preparation of the manuscript by the sponsors.

## Authors’ contributions

DH is the principle investigator of the study, developed study design, drafted the protocol and did the final writing. SB, MH assistant to develop study design, assistant in development protocol, reviewed final writing and gave expert tips. MM assistant to develop study design, reviewed final writing and gave expert tips. CW, US, PG gave expert tips. All authors read and approved the final manuscript.

## Pre-publication history

The pre-publication history for this paper can be accessed here:

http://www.biomedcentral.com/1472-6920/14/32/prepub
